# The Fucosylation Inhibitor, 2-Fluorofucose, Inhibits Vaso-Occlusion, Leukocyte-Endothelium Interactions and NF-ĸB Activation in Transgenic Sickle Mice

**DOI:** 10.1371/journal.pone.0117772

**Published:** 2015-02-23

**Authors:** John D. Belcher, Chunsheng Chen, Julia Nguyen, Fuad Abdulla, Phong Nguyen, Minh Nguyen, Nicole M. Okeley, Dennis R. Benjamin, Peter D. Senter, Gregory M. Vercellotti

**Affiliations:** 1 Department of Medicine, Vascular Biology Center, Division of Hematology, Oncology and Transplantation, Minneapolis, Minnesota, United States of America; 2 Seattle Genetics, Inc., Bothell, Washington, United States of America; Ludwig-Maximilians-Universität, GERMANY

## Abstract

2-Fluorofucose (2FF) blocks the fucosylation and the tethering of sialyl-Lewis^x^ tetrasaccharide and structural variants on leukocytes and red blood cells to P- and E-selectins on activated endothelial cell surfaces. Because P- and E-selectin are required for vaso-occlusion in murine sickle cell disease (SCD), we investigated whether 2FF would inhibit vaso-occlusion in SCD mice. Microvascular stasis was measured in subcutaneous venules in NY1DD and HbSS-Townes SCD mice with dorsal skin-fold chambers after infusion of hemoglobin or exposure to hypoxia/reoxygenation. 2FF in drinking water or administered by gavage inhibited stasis in sickle mice in a dose-responsive manner. Significant inhibitory effects on stasis were seen 1 day post-treatment. 2FF treatment of SCD mice also significantly reduced leukocyte rolling and adhesion along the vessel walls of SCD mice and the static adhesion of neutrophils and sickle red blood cells isolated from 2FF-treated SCD mice to resting and activated endothelial cells. Total white blood cell counts increased in response to 2FF. NF-ĸB activation and VCAM-1 and E-selectin expression were inhibited in the livers of SCD mice consistent with an overall decrease in vascular inflammation and ischemia-reperfusion physiology. Pretreatment with 2FF completely eliminated heme-induced lethality in HbSS-Townes mice, consistent with the observed anti-inflammatory and anti-adhesive properties of 2FF in SCD mice. These data suggest that 2FF may be beneficial for preventing or treating vaso-occlusive crises in SCD patients.

## Introduction

Leukocytes roll along vessel walls by binding to P- and E-selectins on endothelial cell surfaces. The primary leukocyte counter receptors for P- and E-selectin are sialyl-Lewis^x^ (SLe^x^) tetrasaccharide and structural variants that are found on P-selectin glycoprotein ligand-1 (PSGL-1) and E-selectin ligand-1 (ESL-1) [1,2]. These adhesion molecules are required for the initial attachment of leukocytes to the vessel wall and inflammatory extravasation [2–4]. SLe^x^ or a structural variant may also contribute to the adhesion of normal and, to a greater extent, sickle red blood cells (SS-RBC) to endothelial P-selectin [5]. The SLe^x^ counter-receptors contain O-linked fucose molecules that are required for binding to P- and E-selectin [2,6–8]. Because of the important role that fucose plays in adhesion, specific inhibitors of its incorporation could have therapeutic applications. One such inhibitor, 2FF, was recently reported to block fucosylation of SLe^x^ [7,8] and inhibit binding of neutrophils to P- and E-selectin in mice following drinking water administration [8]. This was demonstrated using FACS analysis of neutrophil binding to recombinant mouse E- and P-selectin chimeras and was dose-dependent with maximal inhibition occurring in neutrophils isolated from mice administered 100 mM 2FF in drinking water [8]. Adhesion of blood cells to P- and E-selectin on endothelium plays an essential role in vaso-occlusion in SCD. Knockout of P- and E-selectin or infusion of blocking IgG to P-selectin, E-selectin or SLe^x^ inhibits leukocyte and red blood cell interactions with endothelium and vaso-occlusion in transgenic sickle mice [5,9–13]. Therefore, we tested whether 2FF would inhibit vaso-occlusion, leukocyte rolling and adhesion, and markers of inflammation in SCD mice. Here we report that oral 2FF inhibits vaso-occlusion, interactions of leukocytes and sickle red blood cells with endothelium, NF-ĸB activation and adhesion molecule expression in transgenic sickle mice.

## Methods

### Mice

All animal experiments were approved by the University of Minnesota’s Institutional Animal Care and Use Committee. We utilized male and female NY1DD [14] and HbSS-Townes [15] transgenic sickle mice, age 8–12 months, with weights between 20 and 30g, housed in SPF cages on a 12 hour light/dark cycle at 21°C. All animals were monitored daily including weekends and holidays for health problems, food and water levels and cage conditions. The NY1DD and HbSS-Townes mice are on C57BL/6 and mixed genetic backgrounds, respectively. The NY1DD mice are homozygous for deletion of the mouse β^major^ globin and express a human α and β^S^ globin transgene. NY1DD mice have no anemia, a red blood cell (RBC) half-life of 7 days and a mild disease phenotype. The HbSS-Townes mice were created by knocking in human α and β^S^ globins into the sites where murine α and β globins were knocked out. HbSS-Townes mice have severe anemia, an RBC half-life of 2.5 days and a severe disease phenotype [16]. Littermates were randomly assigned to different treatment groups with equal number of males and females in each treatment group. Different treatment groups were assessed experimentally at the same time. All animals were included in each endpoint analysis and there were no unexpected adverse events that required modification of the protocol.

### Oral administration of 2FF

The 2FF used in these studies has been previously described [8] and was obtained from Seattle Genetics (Seattle, WA). Mice were given the indicated concentrations of 2FF in their drinking water ad libitum or twice daily by gavage (.01 ml/g body weight) for the indicated number of days. 2FF appeared to be well tolerated; none of the mice administered 2FF experienced any infections or death during treatment.

### Dorsal skin-fold chamber (DSFC) implantation

DSFCs were implanted onto sickle mice as previously described [17]. Anesthesia was administered to all mice before surgery and intravital microscopy. The anesthetic cocktail, administered intraperitoneally, consisted of 0.2 ml of xylazine (20 mg/ml) and 0.6 ml of ketamine (100 mg/ml) mixed with 9 ml of normal saline. We injected 0.2–0.4 ml of this anesthetic cocktail into each mouse. All controlled substances used were approved by Research Animal Resources at the University of Minnesota. After the mouse was anesthetized, a suitable location for chamber placement was selected and the hair on that location was shaved off with a clipper. The remaining hair was removed from the dorsal skin with Nair Hair Remover (Church & Dwight, Princeton, NJ). The smooth skin was washed twice with betadine and dried. A sterile field was maintained throughout the surgical procedure. One sterilized chamber piece containing a round window was placed on one side of the dorsal skin fold. Another matching chamber piece without a window was placed on the opposite side of the skin fold, holes for three connecting screws were made through the skin fold and the assembly was tightened together with screws and nuts. A circle of cutaneous tissue and fascia was carefully cut away from the skin fold inside the DSFC window exposing the blood vessels of the subcutaneous tissue adjacent to the striated muscles of the opposing skin fold. Antibiotic ointment (bacitracin zinc/polymyxin B sulfate/neomycin sulfate) was used on the edges of the wound inside the DSFC window. A glass window was placed in the chamber to cover the exposed tissue and secured with a snap ring. Finally, the chamber was sutured to the skin. After surgery, the animals were housed in barrier cages inside a humidified incubator maintained at 32°C with unrestricted access to food and water. All mice were given buprenorphine (0.05–0.1 mg/kg ip) immediately post-DSFC implantation, which was 3 days prior to stasis measurements. To minimize the risk of infection, all mice were given the antibiotic amoxicillin in their drinking water (250 mg per 5 ml water) after DSFC implantation and throughout the experiment.

### Intravital microscopy and measurement of stasis

Three days after DSFC implantation, anesthetized sickle mice were placed on a specially constructed intravital microscopy stage where 20–30 individual flowing venules in each mouse DSFC window were selected at random and their relative locations were noted with a hand drawn map of the microscopic field. After selection and mapping of flowing venules at baseline, mice were given a bolus infusion of stroma-free hemoglobin (Hb) via the tail vein or exposed to 1 hour of hypoxia (7% O_2_/93% N_2_) followed by reoxygenation in room air to induce stasis [13,18]. The same flowing venules selected at baseline were re-examined for stasis (no flow) 1 hour after Hb infusion or after 1 hour of hypoxia and 1 hour of re-oxygenation in room air (H/R). The percent stasis was calculated for each mouse. The sample size (n = 4 mice/treatment) was calculated assuming an effect of 22%, a standard deviation of 10%, a significance level of 5% and a power of 90%.

### Leukocyte rolling and adhesion

Leukocyte rolling and adhesion were measured by intravital microscopy using NY1DD sickle mice (n = 4) with DSFCs as described [17–19]. For the measurement of leukocyte rolling and adhesion, leukocytes were labeled *in vivo* with 100 μL of 0.02% rhodamine-6G (Sigma-Aldrich) in sterile saline solution administered intravenously via tail vein 10 minutes before intravital microscopy. At baseline, flowing venules (n = 5/mouse) were selected at random and their relative locations noted on a map of the microscopic field. Leukocyte-endothelium interactions inside the venular lumen at baseline were captured on camera and saved as digital video files. After collection of baseline videos, the same mice were infused with stroma-free Hb (3.2 μmols heme/kg) via tail vein. One hour after Hb infusion, videos were recorded at the same venule locations that were recorded at baseline. Rolling leukocytes were counted off-line using the video files and were defined as leukocytes that distinctly roll along the endothelial surface of venules. The rolling flux was determined as the total number of leukocytes rolling through a given section of vessel per minute. To assess leukocyte adhesion, we examined 100-μm venular segments and considered a leukocyte adherent if it remained stationary for at least 30 seconds. The sample size (n = 20 venules/treatment) was calculated assuming an effect of 20%, a standard deviation of 20%, a significance level of 5% and a power of 90%.

### Isolation and labeling of murine neutrophils (PMNs) and SS-RBCs

HbSS-Townes mice were administered drinking water with or without 100 mM 2FF for 7 days. Heparinized whole blood was collected by heart puncture and PMNs and SS-RBCs were separated from mononuclear cells as previously described [20] on Histopaque 1077 (Corning Cellgro). The PMNs and SS-RBCs were separated by sedimentation of SS-RBCs on 6% hetastarch (B. Braun Medical) for 90 minutes at room temperature. The remaining SS-RBCs in the PMN fraction were lysed in ice cold water for 30 seconds. PMNs and sedimented SS-RBCs were washed twice in cell culture media containing RPMI 1640, 10% fetal bovine serum, 2 mM L-glutamine and 2 mM sodium pyruvate. Viability of the cell preparation was assessed using trypan blue dye exclusion and was always found to be in excess of 97%. PMNs were fluorescently labeled with 10 mM rhodamine 6G in PBS for 5 minutes at room temperature in the dark. SS-RBCs were fluorescently labeled for 5 minutes in the dark using a red fluorescent cell linker kit for general cell membrane labeling (Sigma-Aldrich #PKH26GL) according to the manufacturer’s protocol. After labeling, PMNs and SS-RBCs were washed 2 times in PBS.

### Endothelial cell culture

Primary human umbilical vein endothelial cells (HUVEC) were cultured to confluence in 96-well plates as previously described [21]. HUVEC were positively identified by co-staining for von Willebrand factor, VE-cadherin, and CD31. For the binding experiments described below, cells were placed in media containing RPMI 1640, 2 mM L-glutamine, 2 mM sodium pyruvate and 1% fetal bovine serum.

### Binding of PMNs and SS-RBCs to HUVEC

Fluorescently labeled PMNs and SS-RBCs isolated from HbSS-Townes mice given drinking water or 100 mM 2FF in drinking water for 7 days were incubated with resting or activated HUVEC. Activated HUVEC, which express P-selectin on their surface, were prepared by incubating HUVEC with 10 μM heme for 30 minutes as previously described [13] prior to addition of PMNs or SS-RBCs. Without further washing of HUVEC, PMNs (20,000 cells/well) or SS-RBCs (1% hematocrit) were added to 4 wells for each condition. The 96-well plates were centrifuged at 200g for 10 minutes and incubated at 37°C for 30 minutes. After 30 minutes, HUVEC were washed 3 times and fluorescent PMNs or SS-RBCs remaining bound to HUVEC were visualized using fluorescence microscopy and digitally photographed at a magnification of 40X. Six fields in each well were randomly selected and photographed. The percent area in each HUVEC field covered with PMNs or SS-RBCs were calculated using Photoshop software (Adobe).

### White blood cell counts

Blood was collected from mice via cardiac puncture into sodium EDTA tubes at the time of euthanasia after administration of 2FF in the drinking water for 7 days. Complete blood counts, differential and hematocrit were measured in EDTA blood as previously described [22]. The sample size (n = 8 mice/treatment) was calculated assuming an effect of 5%, a standard deviation of 3.5%, a significance level of 5% and a power of 90%.

### NF-ĸB activation and adhesion molecules

NF-ĸB p65 (Cell Signaling #3034) and phospho-p65 (Ser536, Cell Signaling #3031) were measured in liver nuclear extracts by western blot as described [23]. Nuclear NF-ĸB phospho-p65 is a specific marker of NF-ĸB activation [24]. VCAM-1 (Abcam, #174279), E-selectin (Boster Immunoleader, #PA1704) and GAPDH (Sigma-Aldrich, #G9545) were measured in liver microsomes by western blot using rabbit primary antibodies and goat anti-rabbit IgG conjugated to alkaline phosphatase (Santa Cruz, #SC-2007). Immunoreactive bands were visualized with ECF substrate (GE Healthcare) and a Storm Reader (GE Healthcare).

### Heme-induced lethality in HbSS mice

HbSS mice (n = 6 mice/treatment) were given a bolus infusion of heme (32 μmols/kg) at time zero. One treatment group received 100 mM 2FF in their drinking water for 7 days prior to heme infusion. Time of death after heme infusion was recorded. Humane endpoints were used during the survival study; mice were euthanized in CO_2_ atmosphere if they became moribund and unable to obtain feed or water. Moribund state was measured by a lack of sustained purposeful response to gentle stimuli. These guidelines were adapted from the American College of Laboratory Medicine and the American Veterinary Medical Association Panel on Euthanasia. Animals were monitored hourly. Signs of pain or distress included decreased activity, abnormal posture, hunched back, muscle flaccidity or rigidity. The sample size (n = 6 mice/treatment) was calculated assuming an incidence of 85% death in the water group and 10% in the 2FF group, a significance level of 5%, and a power of 80%.

### Statistics

Analyses were performed with SigmaStat 3.5 for Windows (Systat Software). Comparisons of multiple treatment groups were made using one-way analysis of variance (ANOVA) (Holm-Sidak method). For comparing two groups, an unpaired *t*-test was used for groups with normal distributions and a Mann-Whitney Rank Sum Test was used for groups that failed the normality test.

## Results and Discussion

### 2FF inhibits microvascular stasis in transgenic sickle mice

**Fig 1:**
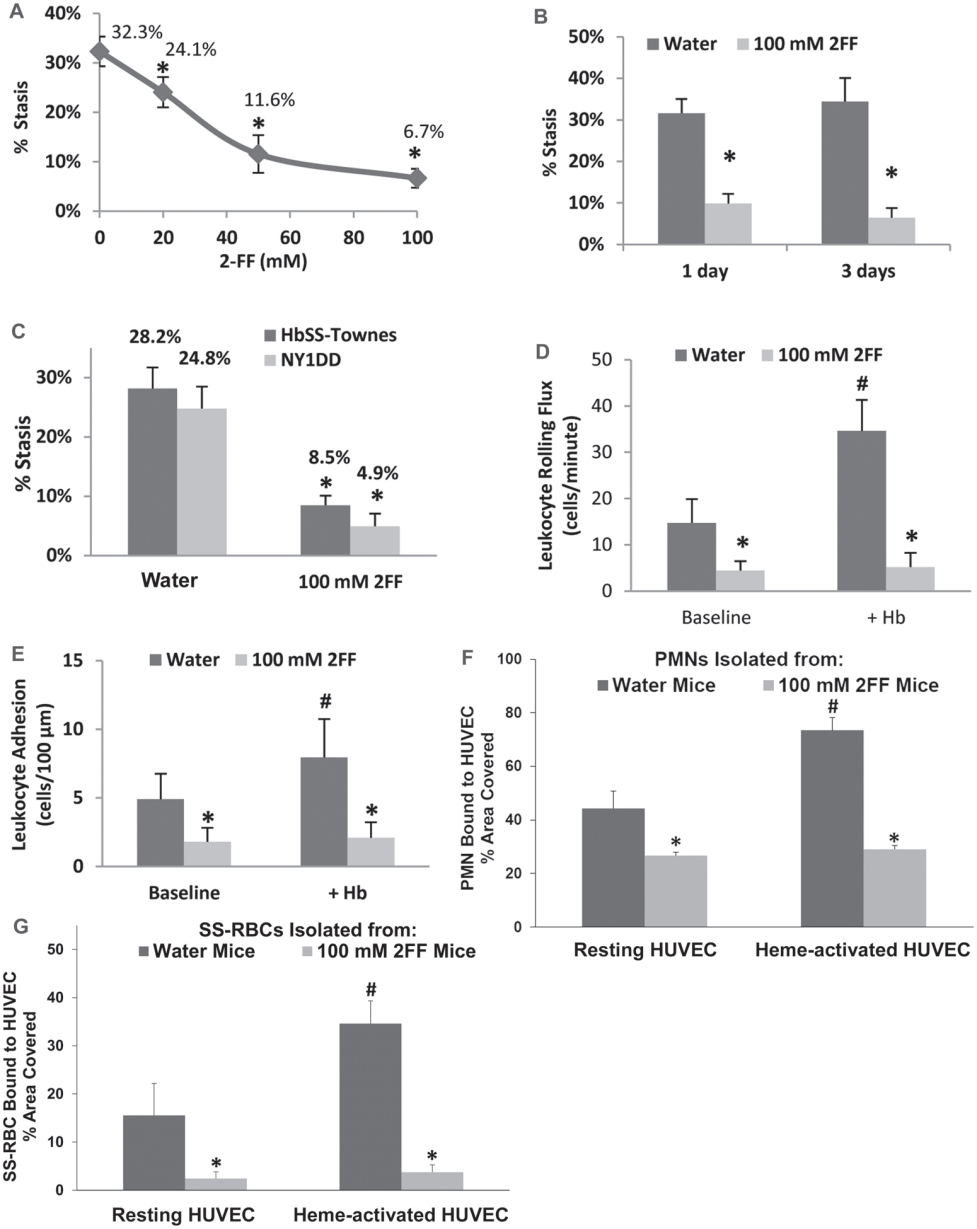
2FF inhibits microvascular stasis and leukocyte rolling and adhesion in sickle mice *in vivo*. (**A**) NY1DD transgenic sickle mice (n = 4/group) were given the indicated concentration of 2FF in their drinking water for 7 days. Microvascular stasis was measured on day 7, one hour after the infusion of stroma-free Hb (3.2 μmols heme/kg). *P≤.05 for all pairwise comparisons. (**B**) NY1DD sickle mice were gavaged with water or 150 mg/ml 2FF (.01 ml/g, twice per day) for 1 or 3 days. After the indicated 2FF treatment period, stroma-free Hb (3.2 μmols heme/kg body weight) was infused intravenously and microvascular stasis was measured 1 hour later. *P≤.01 for water vs 2FF. (**C**) NY1DD and HbSS-Townes sickle mice (n = 3/group) were given 100 mM 2FF in their drinking water for 7 days. Stasis was measured on day 7 after exposure to 1h of hypoxia and 1h of reoxygenation (H/R). *P<.001 for water vs 100 mM 2FF. (**D**) Leukocyte rolling flux was measured in venules in the DSFC windows of NY1DD mice before (baseline) and 1h after infusion of Hb (3.2 μmol/kg). Half of the mice (n = 4) were treated with 2FF (100 mM in drinking water X 7 days) prior to baseline measurements. Control mice (n = 4) were given drinking water without 2FF. The rolling flux was determined as the total number of leukocytes rolling through a given section of vessel per minute. Values are means + SD. ^**#**^P<.001 baseline vs Hb and *p<.001 for water vs 2FF. (**E**) Leukocyte adhesion was measured in the same venules as described in (D). Values are mean number of adherent cells per 100 μm + SD. ^**#**^P<.001 for baseline vs Hb and *p<.001 for water vs 2FF. (**F** and **G**) PMNs (**F**) and SS-RBCs (**G**) were isolated from HbSS-Townes mice (n = 2 mice twice) given drinking water or 100 mM 2FF in drinking water for 7 days. Fluorescently labeled PMNs or SS-RBCs were incubated with resting and activated HUVEC (4 wells/treatment) for 30 minutes. Activated HUVEC, which express P-selectin on their surface, were prepared by incubating HUVEC with 10 μM heme for 30 minutes. Values are mean % area of HUVEC cell surface covered by PMNs (**F**) or SS-RBCs (**G**) + SD after HUVEC washing. ^**#**^P<.001 for resting vs activated and *p<.001 for water vs 2FF.

Microvascular stasis was measured in subcutaneous venules in NY1DD sickle mice with implanted DSFCs after infusion of stroma-free Hb. 2FF administered in the drinking water for 7 days inhibited Hb-induced stasis in NY1DD sickle mice (see the structure of 2FF in S1 Fig.). Stasis decreased as the concentration of 2FF in the drinking water increased (Fig. 1A). Significant levels of inhibition were seen at 20, 50 and 100 mM 2FF (p≤.05). When 2FF was administered to NY1DD mice by gavage (150 mg/ml X. 01 ml/g body weight X twice/day), significant decreases in Hb-induced microvascular stasis were seen after 1 and 3 days of 2FF gavage compared to mice gavaged with water (p<.01, Fig. 1B). Stasis induced by H/R was also inhibited in NY1DD and HbSS-Townes sickle mice treated with 100 mM 2FF in the drinking water for 7 days compared to water (p<.001, Fig. 1C).

### Leukocyte rolling and adhesion are inhibited in 2FF-treated sickle mice

Administration of 100 mM 2FF to mice in drinking water for 7 days blocks fucosylation of SLe^x^ [7,8] and inhibits binding of neutrophils (PMNs) to recombinant P- and E-selectin chimeras [8]. To further extend these observations, leukocyte rolling and adhesion were measured in subcutaneous venules in NY1DD sickle mice with implanted DSFCs at baseline and after infusion of stroma-free Hb. At baseline, immediately before Hb infusion, leukocyte rolling and adhesion were significantly less in sickle mice pretreated with 100 mM 2FF in drinking water for 7 days compared to mice administered water (p<.001, Fig. 1D and 1E and S1 Video). Leukocyte rolling and adhesion increased significantly one hour after Hb infusion in sickle mice given drinking water (p<.001), but not in animals administered 2FF for 7 days in the drinking water (Fig. 1D and 1E and S1 Video). Leukocyte rolling and adhesion were significantly less after infusion of Hb in sickle mice administered 2FF compared to water controls (p<.001).

### Adhesion of 2FF-treated PMNs and SS-RBCs to HUVEC is inhibited

PMNs and SS-RBCs were isolated from HbSS-Townes mice given drinking water or 100 mM 2FF in drinking water for 7 days. Fluorescently labeled PMNs and SS-RBCs were incubated with resting and activated HUVEC for 30 minutes. Activated HUVEC, which express P-selectin on their surface, were prepared by incubating HUVEC with 10 μM heme for 30 minutes [13]. The binding of PMNs or SS-RBCs from water-treated sickle mice, but not 2FF-treated sickle mice, was significantly greater to heme-activated HUVEC than binding to resting HUVEC (p<0.001, Fig. 1F and 1G). The number of PMNs or SS-RBCs bound to resting or activated HUVEC was significantly lower in mice administered 100 mM 2FF in drinking water compared to water controls (p<0.001, Fig. 1F and 1G). The binding of PMNs and SS-RBCs from 2FF-treated animals was not significantly different between activated and resting HUVEC (Fig. 1F and 1G). These results are consistent with the decreases in vaso-occlusion seen in Fig. 1A–C and the decrease in leukocyte rolling and adhesion along the vessel wall seen in Fig. 1D and E.

Inhibition of leukocyte adhesion to the vessel wall or inhibition of platelet/leukocyte/red blood cell heterotypic aggregates is sufficient to prevent vaso-occlusion in murine SCD models [9,10,12,13,25–28]. RBC half-lives in our NY1DD model is ~7 days (unpublished data) while the reported PMN half-life is 11 hours in mice [29]. The rapid response to 2FF in SCD mice (1 day in Fig. 1B), suggests that inhibition of leukocyte fucosylation may be responsible for the observed effects on vaso-occlusion after 1 day of treatment with 2FF.

### 2FF increases the white blood cell (WBC) count in sickle mice

Two separate groups of NY1DD sickle mice were given water, 20 mM or 100 mM 2FF in drinking water for 7 days. After 7 days of 20 or 100 mM 2FF in the drinking water, mean plasma levels of 2FF were 67 and 127 μM in the 20 mM treatment groups and 156 and 490 μM in the 100 mM treatment groups (S2 Fig.). WBC counts were measured in the two treatment groups but did not differ significantly between groups so the WBC data from both groups were pooled. 2FF increased total PMN and lymphocyte WBC counts in a dose-dependent fashion (Table 1). The highest WBC counts were seen in sickle mice treated with 100 mM 2FF.

**Table 1:** 2FF increases the total, neutrophil (PMN) and lymphocyte white blood cell (WBC) counts in sickle mice.

Treatment	N	Total WBC		PMN		Lymphocytes	
		Mean	SD	Mean	SD	Mean	SD
**Water**	8	16.3	3.2	4.6	1.5	10.6	2.3
**20 mM 2FF**	8	22.1*	5.2	6.6	2.4	14.3*	3.8
**100 mM 2FF**	8	34.2* ^**#**^	7.2	13.2* ^**#**^	5.2	19.2* ^**#**^	3.5

NY1DD sickle mice were given the indicated concentration of 2FF in drinking water for 7 days. Blood was collected and WBC, PMN and lymphocyte counts were measured on day 7.

*P<.05 for 20 or 100 mM 2FF vs water and

^#^p<.05 for 100 mM 2FF vs 20 mM 2FF. Values are cells/μL X 10^-3^.

### 2FF decreases NF-ĸB activation and adhesion molecule expression in sickle mice

**Fig 2:**
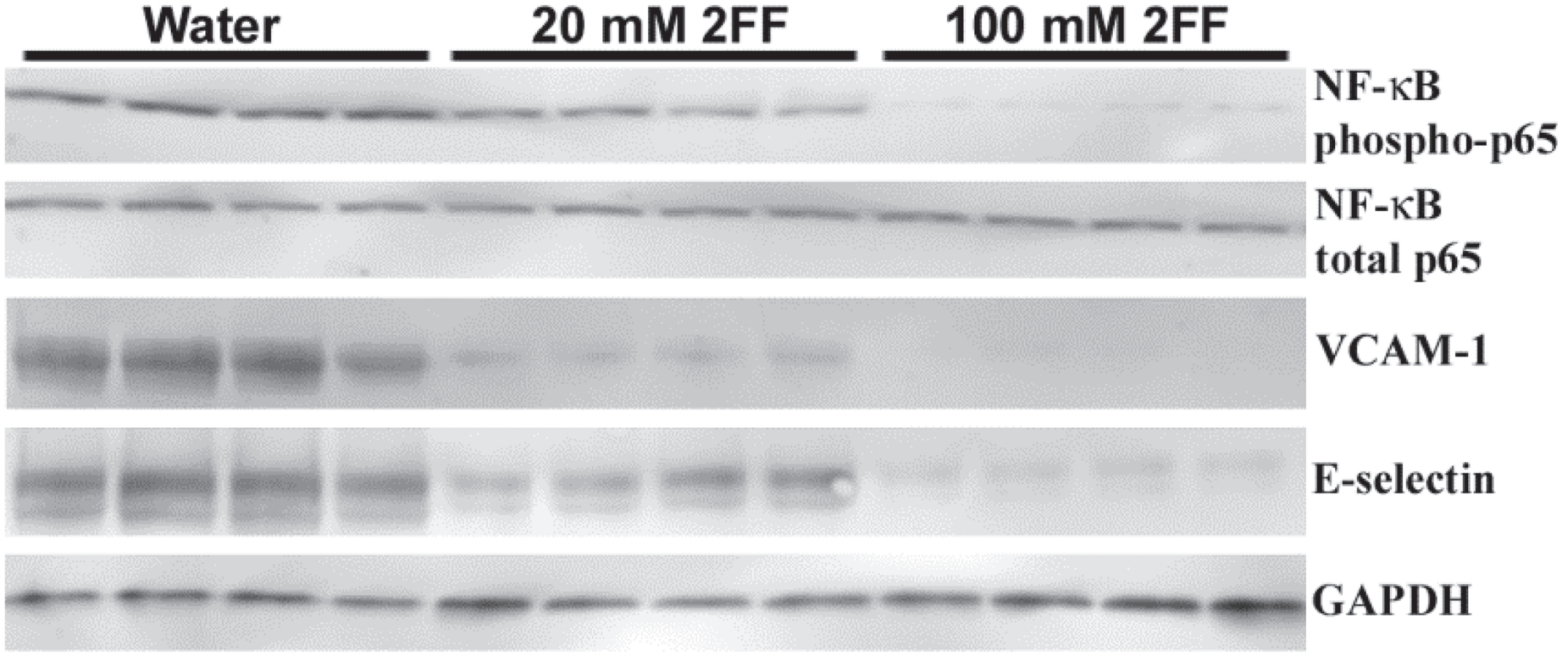
2FF inhibits NF-ĸB activation and adhesion molecule expression in livers of sickle mice. After 7 days administration of water or 100 mM 2FF in drinking water and 4 hours after infusion of stroma-free Hb, livers from NY1DD sickle mice were removed and flash frozen. NF-kB phospho- and total-p65 were measured by western blot using liver nuclear extracts. Nuclear NF-ĸB phospho-p65 is a marker of NF-ĸB activation. VCAM-1, E-selectin and GAPDH were measured by western blot using liver microsomes.

SCD mice mimics ischemia-reperfusion physiology [9,30] as static venules often re-open to flow after several hours of stasis [18] leading to NF-ĸB activation and tissue injury. Stimulation of vaso-occlusion in SCD mice activates endothelial NF-ĸB as indicated by the formation of NF-ĸB phospho-p65 and upregulation of adhesion molecule expression [13,19]. Inhibitors of vaso-occlusion prevent these inflammatory responses. Therefore, we evaluated the effects of 2FF on hepatic NF-κB activation and expression of adhesion molecules under NF-κB control. Livers were collected 4 hours after infusion of Hb in mice given water, 20 mM or 100 mM 2FF in their drinking water for 7 days. Nuclear NF-ĸB phospho-p65, a marker of NF-ĸB activation [24], was decreased in livers as the concentration of 2FF increased (Fig. 2). NF-ĸB phospho-p65 was markedly decreased in mice treated with 100 mM 2FF, while total NF-ĸB p65 did not change significantly in liver nuclear extracts. Similar decreases in NF-ĸB-driven adhesion molecules, VCAM-1 and E-selectin, were seen in the livers of animals treated with 2FF (Fig. 2). Both of these adhesion molecules are required for microvascular stasis in sickle mice [13].

### 2FF rescues HbSS-Townes mice from heme-induced lethality

**Fig 3:**
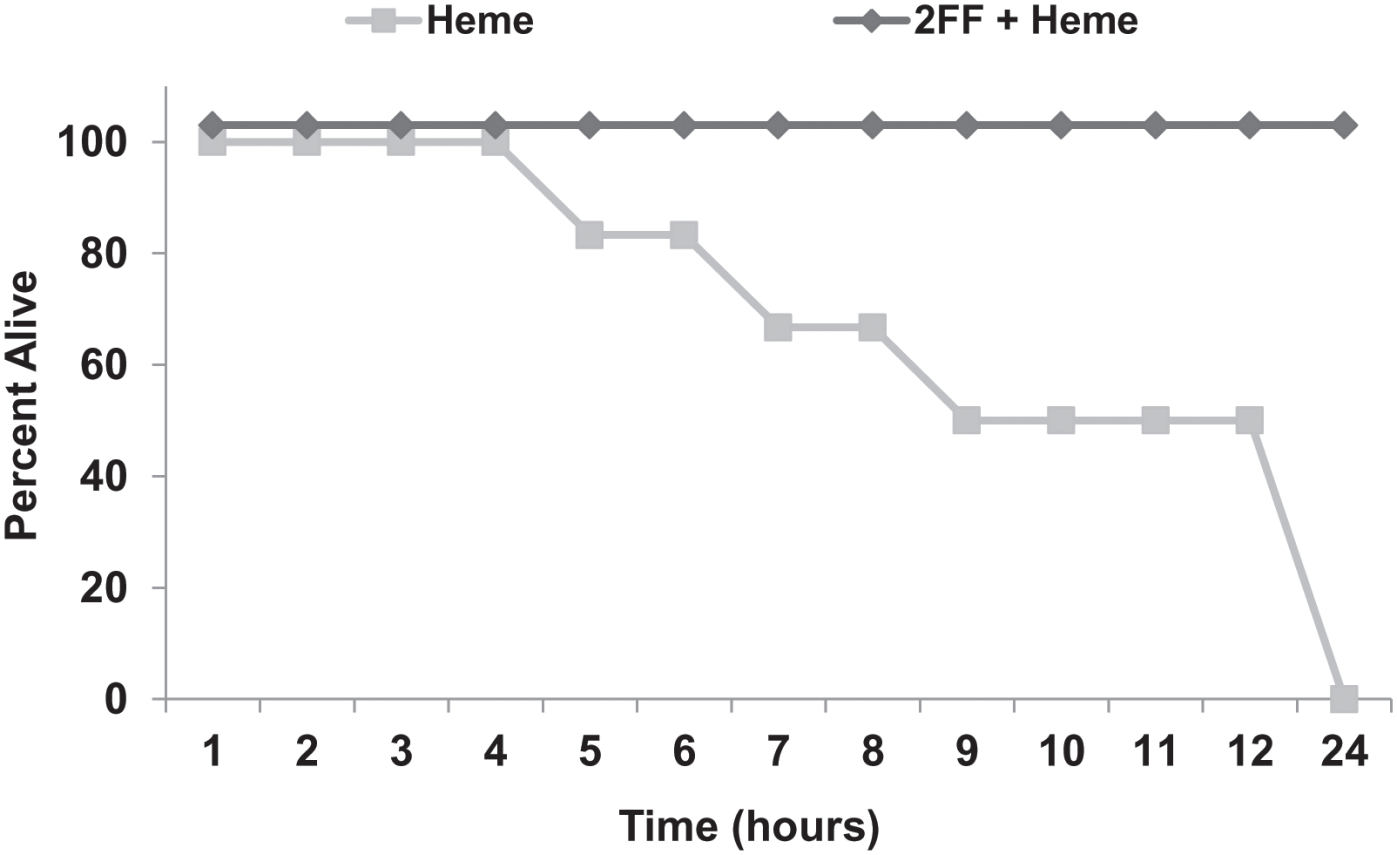
2FF prevents heme-induced lethality in HbSS-Townes mice. All HbSS-Townes mice (n = 6 mice/treatment) were given a bolus infusion of heme (32 μmols/kg) at time zero. Mice were given water or 100 mM 2FF in drinking water for 7 days prior to heme infusion. Time of death after heme infusion was recorded.

Heme induces leukocyte rolling/adhesion, vaso-occlusion and lung and liver injury in HbSS-Townes mice [13,31]. Heme infused into HbSS-Townes mice was lethal within 24 hours (Fig. 3). Pretreatment with 100 mM 2FF for 7 days prevented heme-induced lethality. Although necropsies could not determine a definitive cause of death, inhibition of vaso-occlusion may explain the protection afforded by 2FF.

New treatments that inhibit vaso-occlusive crises (VOC) are urgently needed for treating SCD patients. There is considerable interest in selectin inhibitors. In a Phase 2 trial, SCD patients hospitalized for VOC were treated intravenously with GMI-1070, a synthetic gylcomimetic molecule and pan-selectin inhibitor. Patients treated with GMI-1070 experienced reductions in time to reach resolution of VOC, length of hospital stay and use of opioid analgesics for pain management [32]. In contrast, an oral agent 2FF, an inhibitor of protein fucosylation, is a potent inhibitor of leukocyte-endothelium interactions, vaso-occlusion and NF-ĸB activation in SCD mice that have been challenged with Hb or H/R. There would be considerable advantage having an oral agent like 2FF in treating or preventing VOC. Use of 2FF to treat other inflammatory conditions may also be warranted. Long-term prophylactic use of 2FF in chronic inflammatory conditions such as SCD will need to be balanced against the potential inhibitory effects of 2FF on the host defense response to pathogens.

## Supporting Information

S1 ARRIVE Checklist (PDF)

S1 Fig Structure of 2FF. (PPTX)

S2 Fig Plasma Levels of 2FF. (PPTX)

S1 Video 2FF Inhibits Leukocyte Rolling and Adhesion. (PPTX)
