# Supplementary figures and images for: The Fucosylation Inhibitor, 2-Fluorofucose, Inhibits Vaso-Occlusion, Leukocyte-Endothelium Interactions and NF-ĸB Activation in Transgenic Sickle Mice
Source: PLoS One. 2015 Feb 23;10(2):e0117772. doi: 10.1371/journal.pone.0117772 (PMC4338063; doi:10.1371/journal.pone.0117772)

## Slide 1
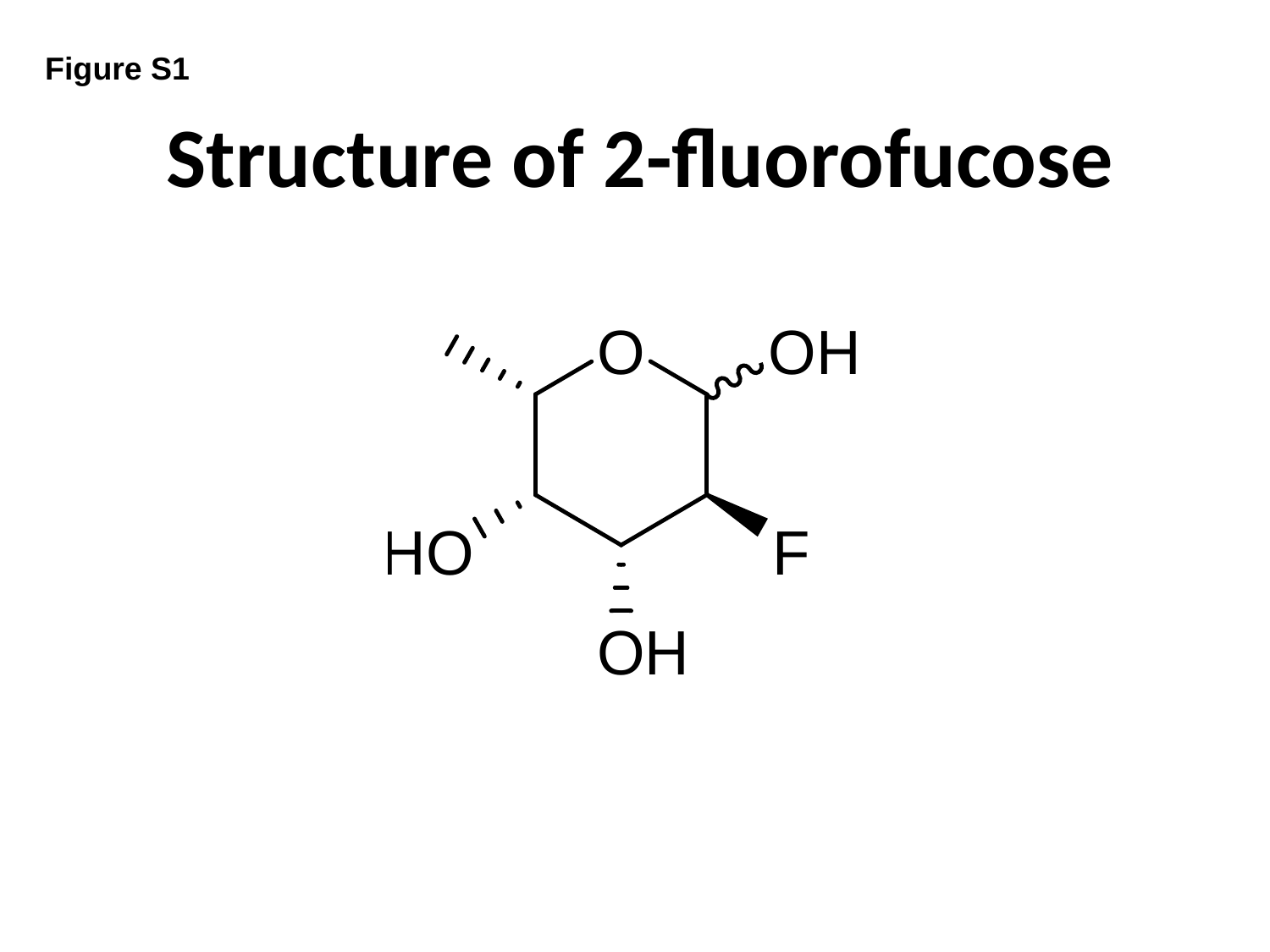

Figure S1
Structure of 2-fluorofucose

Supplement: S1 Fig — (PPTX) [file pone.0117772.s002.pptx]
