# Supplementary material for: The Fucosylation Inhibitor, 2-Fluorofucose, Inhibits Vaso-Occlusion, Leukocyte-Endothelium Interactions and NF-ĸB Activation in Transgenic Sickle Mice
Source: PLoS One. 2015 Feb 23;10(2):e0117772. doi: 10.1371/journal.pone.0117772 (PMC4338063; doi:10.1371/journal.pone.0117772)
Supplement: S2 Fig — (PPTX) [file pone.0117772.s003.pptx]

## Slide 1
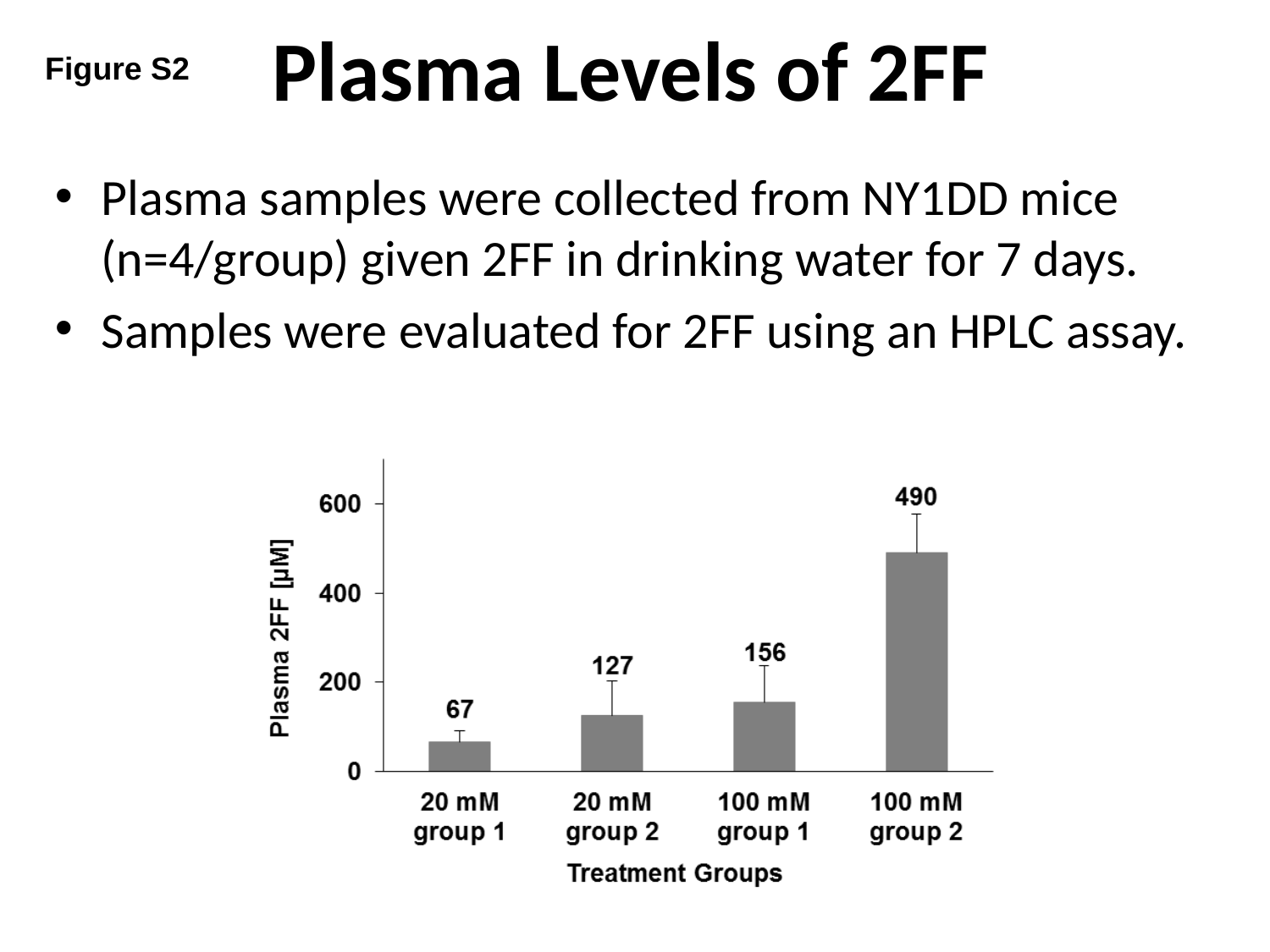

Plasma Levels of 2FF
Figure S2
Plasma samples were collected from NY1DD mice (n=4/group) given 2FF in drinking water for 7 days.
Samples were evaluated for 2FF using an HPLC assay.
