# Supplementary material for: The Fucosylation Inhibitor, 2-Fluorofucose, Inhibits Vaso-Occlusion, Leukocyte-Endothelium Interactions and NF-ĸB Activation in Transgenic Sickle Mice
Source: PLoS One. 2015 Feb 23;10(2):e0117772. doi: 10.1371/journal.pone.0117772 (PMC4338063; doi:10.1371/journal.pone.0117772)
Supplement: S1 Video — (PPTX) [file pone.0117772.s004.pptx]

## Slide 1
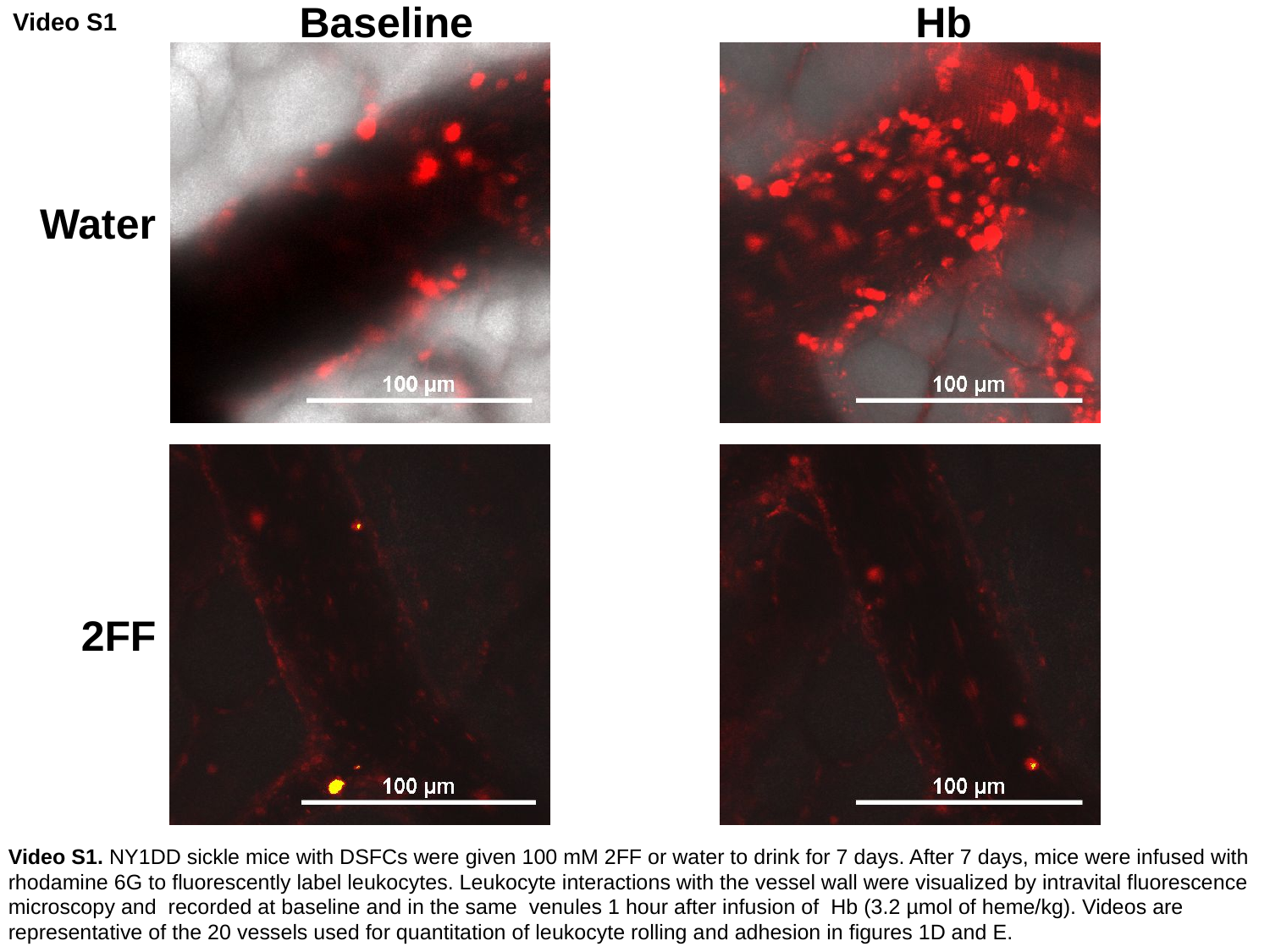

Video S1
Baseline
Hb
Water
2FF
Video S1. NY1DD sickle mice with DSFCs were given 100 mM 2FF or water to drink for 7 days. After 7 days, mice were infused with rhodamine 6G to fluorescently label leukocytes. Leukocyte interactions with the vessel wall were visualized by intravital fluorescence microscopy and recorded at baseline and in the same venules 1 hour after infusion of Hb (3.2 µmol of heme/kg). Videos are representative of the 20 vessels used for quantitation of leukocyte rolling and adhesion in figures 1D and E.
